# Detection of glycoprotein of Burkholderia pseudomallei.

**DOI:** 10.3201/eid0402.980229

**Published:** 1998

**Authors:** N P Khrapova, N G Tikhonov, Y V Prokhvatilova


and the one presented by Jiang (5) suggest that both the SDF-1 chemokine and the V3 loop of T-tropic HIV-1 viruses use positively charged amino acids for an electrostatic interaction with the negatively charged CXCR4 receptor. To examine whether the similarity between the chemokine and env V3 domain is also apparent at the primary sequence level, we performed an amino acid alignment; however, we found no conserved motifs (data not shown). A detailed mutational analysis is required to further our understanding of the env-coreceptor interaction.

**Ben Berkhout and Atze T. Das**
Department of Human Retrovirology, Academic Medical Center, University of Amsterdam, Amsterdam, The Netherlands
(b.berkhout@amc.uva.nl)

### References

1. De Jong J-J, Ronde de A, Keulen W, Tersmette M, Goudsmit J. Minimal requirements for the human immunodeficiency virus type 1 V3 domain to support the syncytium-inducing (SI) phenotype: analysis by single amino acid substitution. J Virol 1992;66:6777-80.
2. Feng Y, Broder CC, Kennedy PE, Berger EA. HIV-1 entry cofactor: functional cDNA cloning of a seven-transmembrane, G protein coupled receptor. Science 1996;272:872-7.
3. Deng H, Liu R, Ellmeier W, Choe S, Unutmaz D, Burkhart M, et al. Identification of a major co-receptor for primary isolates of HIV-1. Nature 1996;381:661-6.
4. Lapham CK, Ouyang J, Chandrasekar B, Nguyen NY, Dimitrov DS, and Golding H. Evidence for cell-surface association between fusin and the CD4-gp120 complex in human cell lines. Science 1996;274:602-5.
5. Jiang S. HIV-1 co-receptors binding. Nat Med 1997;3:367-8.
6. Oberlin E, Amara A, Bachelerie F, Bessia C, Virelizier J-L, Arenzana-Seisdedos F, et al. The CXC chemokine SDF-1 is the ligand for LESTR/fusin and prevents infection by T-cell-line-adapted HIV-1. Nature 1996;382:833-5.
7. Premack BA, Schall TJ. Chemokine receptors: gateways to inflammation and infection. Nat Med 1996;2:1174-8.
8. Cocchi F, DeVico AL, Garzino-Demo A, Arya SK, Gallo RC, Lusso P. Identification of RANTES, MIP-1 alpha and MIP-1 beta as the major HIV-suppressive factors produced by CD8+ T cells. Science 1995;270:1811-5.
9. Amara A, Le Gall S, Schwartz O, Salamero J, Montes M, Loetscher P, et al. HIV coreceptor downregulation as antiviral principle: SDF-1a-dependent internalization of the chemokine receptor CXCR4 contributes to inhibition of HIV replication. J Exp Med 1997;186:139-46.

### Detection of Glycoprotein of *Burkholderia pseudomallei*

**To the Editor**: Melioidosis, a potentially fatal disease that is difficult to diagnose and treat, is common in areas with subtropical climate (e.g., Singapore, the southern provinces of China) and is hyperendemic in Thailand. The etiologic agent, *Burkholderia pseudomallei* (*Pseudomonas pseudomallei*), is widely distributed in Southeast Asia and northern Australia. The agent has the potential to become established in regions with similar climate conditions, particularly if animals infected with *B. pseudomallei* are imported from endemic-disease zones (1-3).

Rapid and reliable detection of *B. pseudomallei* and its antigens has many potential applications. Recently, we developed a monoclonal antibody immunoenzyme test system for the detection of minimal concentrations of a *B. pseudomallei* glycoprotein, which is considered one of the pathogenicity factors for this microorganism. This glycoprotein, called Ag8 by N.N. Piven and V.I. Ilyukhin (4), is present in different strains of *B. pseudomallei* and *B. mallei* but not in other *Burkholderia* spp. (*B. aeruginosa*, *B. putida*, *B. cepacia*, *B. malthophilia*, *B. fluorescens*, *B. pseudoalcaligenes*). Ag8 is composed of 10% protein and 90% carbohydrate, has molecular mass 800 kDa, and is localized in an extracellular capsulelike substance surrounding *B. pseudomallei* cells (5).

We developed an immunoenzyme test system with three monoclonal antibodies (Mab) to different epitopes of Ag8 (Mab 2A6-IgG3, Mab 2H7-IgG1, Mab 1G2-IgG2b) and one antibody to epitopes common for Ag8 and LPS of *B. pseudomallei* (mab 1ES-IgG2b). A sandwich enzyme-linked immunosorbent assay (ELISA) was used for the detection of Ag8 in different test samples (6). The sensitivity of the immunoenzyme test system was determined with a standard antigen sample. Minimal sensitivity (37 ng/ml of carbohydrate) was observed when polyclonal immunoglobulins were used as "catching" antibodies. Maximal sensitivity (0.37 ng/ml of carbohydrate) was noted when either Mabs 2A6 or mixtures of Mabs were used as catching antibodies.

The test system was further evaluated with samples of extracellular antigens (extracts of cultural media, fractions after gel chromatography of extracellular antigens) and bacterial suspensions of *B. pseudomallei* and *B. mallei* strains isolated in different regions of the world.

Levels of Ag8 in cultural media varied considerably depending on periods of cultivation of bacteria. Additionally, the level of Ag8 varied among strains of *B. pseudomallei* and *B. mallei*. Among 61 strains of *B. pseudomallei* from the museum collection (most of which were isolated in Southeast Asia and northern Australia), three had increased ability to produce Ag8. These strains had been isolated from clinical specimens (blood, abscesses of hospitalized melioidosis patients) in Vietnam. The strains gave results typical of *B. pseudomallei* species in all routine serologic tests (agglutination test, immunofluorescence assay, immunodiffusion test). In contrast, the *B. pseudomallei* glanders agent (16 strains from the museum collection) had reduced ability for Ag8 production; ELISA titers of Ag8 were a thousandfold less in culture fluids in these strains.

The ELISA technique not only facilitates diagnosis of disease but also provides a rational basis for selecting strains for vaccine production. It also has considerable utility for studying the pathogenicity of *B. pseudomallei*.

**Natalja P. Khrapova, Nikolay G. Tikhonov, Yelena V. Prokhvatilova**
Volgograd Antiplague Research Institute, Volgograd, Russia

### References

1. Dodin A. La mélioidose: un probleme mondial. Recherche 1976;7(68):574-7.
2. Galimand M, Dodin A. Le point sur la melioidose dans le monde. Bull Soc Pathol Exot 1982;75,4:375-83.
3. Larionov GM, Pogasiy NI. A methodology for determination of reservoirs of the causative agents of sapronoses. Med Parazitol (Mosk) 1995;4:45-9.
4. Piven NN, Ilyukhin VI. Antigenic structure of *Pseudomonas pseudomallei*. Zh Microbiol Epidemiol Immunobiol 1981;4:78-82.
5. Kapliev VI, Piven NN, Khrapova NP. Determination of antigens on melioidosis agents with the use of monoclonal antibodies at the ultrastructural level. Mikrobiol Z 1992;54,1:85-9.
6. Voller A, Bidwell DE, Bartlett A. Enzyme immunoassays in diagnostic medicine. Bull World Health Organ 1976;53,1:55-65.

### Forging New Perspectives on Disease Surveillance

**To the Editor:** Recognizing disease emergence as a paradigm uniquely influenced by human activity demands a reevaluation of traditional disease surveillance systems. Part of a surveillance program should be focused on the areas of human activity where disease emergence is most likely to occur. A system that monitors areas known to be involved in disease emergence, such as development projects, agriculture, climate, and refugee movements, may greatly increase our ability to detect and prevent outbreaks.

Large development projects entail ecologic upheavals that can facilitate disease emergence. Construction of a dam in 1987 in Mauritania resulted in increased mosquito breeding sites and in an explosion in the mosquito population; epidemics of Rift Valley fever quickly followed (1). The Southeastern Anatolia Irrigation Project on the Euphrates and Tigris Rivers in Turkey, which will provide irrigation for 1.7 million hectares, has already increased malaria and leishmaniasis cases in the local population (2). The massive Three Gorges Dam Project on the Yangtze River in China, which will create a reservoir 760 km long, must be evaluated for its impact on local disease. With knowledge of endemic diseases and their reservoirs and vectors in these areas of ecologic change, public health workers can anticipate disease epidemics and implement prevention measures.

Incorporating climate predictions into a disease surveillance system would supplement resources in an area known to affect disease emergence. The U.S. Agency for International Development's (USAID) Famine Early Warning System monitors the African continent for two major factors implicated in emergence: temperature and precipitation. Focused on countries at high risk for food shortages and famine, the early warning system is an example of a predictive and preventative surveillance system. Precipitation, temperature, and plant health data from satellites are evaluated as indicators of crop failure. These data are supplemented by information from field representatives who directly observe agricultural production. USAID's system and other global monitoring systems can provide a base level of surveillance that can add to our knowledge of climatologic influence on disease emergence.

The beginning of the Zairian refugee crisis in 1994 illustrates the need for surveillance among refugee populations. In July 1994, 500,000 to 800,000 Rwandan Hutus fled into the North Kivu region of Zaire. In the month between July 14 and August 14, 48,347 of these refugees died,

